# The long-term impact of the COVID-19 pandemic on primary and specialized care provision and disease recognition in Germany

**DOI:** 10.3389/fpubh.2022.1006578

**Published:** 2022-11-17

**Authors:** Moritz Platen, Jens Bohlken, Wolfgang Hoffmann, Karel Kostev, Bernhard Michalowsky

**Affiliations:** ^1^German Center for Neurodegenerative Diseases (DZNE), Greifswald, Germany; ^2^Institute for Social Medicine, Occupational Medicine, and Public Health (ISAP) of the Medical Faculty, University of Leipzig, Leipzig, Germany; ^3^Institute for Community Medicine, Section Epidemiology of Health Care and Community Health, University Medicine Greifswald (UMG), Greifswald, Germany; ^4^Epidemiology, IQVIA, Frankfurt am Main, Germany

**Keywords:** COVID-19, healthcare utilization, disease recognition, hospitalization, primary care

## Abstract

**Background:**

The COVID-19 pandemic and the imposed lockdowns severely affected routine care in general and specialized physician practices.

**Objective:**

To describe the long-term impact of the COVID-19 pandemic on the physician services provision and disease recognition in German physician practices and perceived causes for the observed changes.

**Design:**

Observational study based on medical record data and survey data of general practitioners and specialists' practices.

**Participants:**

996 general practitioners (GPs) and 798 specialist practices, who documented 6.1 million treatment cases for medical record data analyses and 645 physicians for survey data analyses.

**Main measures:**

Within the medical record data, consultations, specialist referrals, hospital admissions, and documented diagnoses were extracted for the pandemic (March 2020–September 2021) and compared to corresponding pre-pandemic months in 2019. The additional online survey was used to assess changes in practice management during the COVID-19 pandemic and physicians' perceived main causes of affected primary and specialized care provision.

**Main results:**

Hospital admissions (GPs: −22% vs. specialists: −16%), specialist referrals (−6 vs. −3%) and recognized diseases (−9 vs. −8%) significantly decreased over the pandemic. GPs consultations initially decreased (2020: −7%) but compensated at the end of 2021 (+3%), while specialists' consultation did not (−2%). Physicians saw changes in patient behavior, like appointment cancellation, as the main cause of the decrease. Contrary to this, they also mentioned substantial modifications of practice management, like reduced (nursing) home visits (41%) and opening hours (40%), suspended checkups (43%), and delayed consultations for high-risk patients (71%).

**Conclusion:**

The pandemic left its mark on primary and specialized healthcare provision and its utilization. Both patient behavior and organizational changes in practice management may have caused decreased and non-compensation of services. Evaluating the long-term effect on patient outcomes and identifying potential improvements are vital to better prepare for future pandemic waves.

## Introduction

Due to the COVID-19 pandemic and its rapid spread, governments worldwide initiated emergency lockdowns, mitigating infection rates and preventing the burden on the healthcare systems (1). Social distancing has become the standard practice in preventing the spread of COVID-19 infection (1–3).

Measures mitigating the infection rates resulted in side effects on the provision and utilization of routine care in worldwide healthcare systems. Healthcare utilization was affected due to efforts of reduced infection risk to prevent the older population and the overburden of the healthcare system (4). However, the infection risk and mortality rate also affected the provision of healthcare services during the pandemic, especially for general practitioners (GPs) (5).

Previous studies report that, initially, primary care physicians were unprepared for the new situation in their practices, especially regarding medical equipment, leading to significant concerns and fears among physicians, including being carriers of the virus or becoming infected (6, 7). GPs were concerned about the continuity of regular care due to COVID-19 measures and that these could affect the population's overall health (8). It can be assumed that the lack of medical equipment and the fear reflected in physicians' behavior entailed adjusting their practice management. However, evidence about related consequences at the individual practice level affecting healthcare provision is rare. Therefore, primary data are needed to fill this gap.

Despite several studies examining the impact of early imposed contact bans on the provision and utilization of primary and secondary healthcare services, indicating a tremendous decrease in physician consultations, specialist referrals, hospital admissions and disease recognition, there is presently a lack of knowledge about the compensation of the initial decline during the following course of the COVID-19 pandemic (9–14). Moreover, analyses of the causal background of changes in health service provision and the lack of compensation beyond the early COVID-19 contact bans are scarce.

Therefore, this study aimed to describe (i) the provision of healthcare services and disease recognition during the COVID-19 pandemic in Germany's primary and specialized care and (ii) to identify reasons for the change of both in the following course of the pandemic from an outpatient physician perspective.

## Materials and methods

### Study design

This study comprised a secondary data analysis to examine changes in healthcare service provision and an additional survey to assess perceived reasons for the changes in primary and specialized care provision.

The secondary data analysis was based on medical record data from the Disease Analyzer database (IQVIA), capturing consultations, drug prescriptions, specialist referrals, diagnoses made, and basic medical and demographic data directly and anonymously from the primary care and specialist practices *via* an interface to their respective practice management software (15). The database structure corresponds to the total number of physicians (i.e. statistical population) annually published by the German Medical Association in terms of demographic characteristics, diagnoses, and therapies for each specialty and covers about 3% of all outpatient practices in Germany (15). Diagnoses, prescriptions, and quality of the reported data are monitored by IQVIA using several criteria, such as completeness and plausibility (15). The analysis was based on data from a total of 6.1 million treatment cases per year (patients aged 18 years and older) documented by 996 GPs and internists or 798 specialist practices (*n* = 224 gynecologists, *n* = 147 orthopedists, *n* = 127 neurologists and psychiatrists, *n* = 133 ear, nose and throat (ENT) physicians, *n* = 83 dermatologists, and *n* = 84 urologists) in Germany between September 2019 and February 2020 (pre-pandemic period) as well as March 2020 and September 2021 (pandemic period).

The subsequent Germany-wide anonymous online survey was generated based on the previously conducted secondary data analysis results, daily developments, and internal expert consensus and comprised, in particular, the following steps: (i) review of the relevant literature, (ii) development of the first draft of the questionnaire by a previously formed core team of three researchers, (iii) consultation of the questionnaire with representatives (i.e. experts) of all participating professional associations as well as (iv) finalization and preparation for distribution. The questionnaire was distributed using the cloud-based open-source tool LimeSurvey (16). The following professional associations of practitioners shared the survey link between 04 December 2021 and 28 February 2022 with their members via different communication channels (e.g., e-mail distribution lists, newsletters, and homepages): German General Practitioners Association e.V., Professional Association of German Neurologists, Professional Association of German Urologists e.V., German Professional Association of Otolaryngologists e.V. Participating primary care physicians and specialists were informed about the changes in healthcare utilization during COVID-19 by the results of the secondary data analysis and were subsequently asked to fill in the questionnaire about their perceptions and reasons for the change in the provision and utilization of primary and specialized healthcare services. In total, *n* = 645 physicians responded to the online survey questionnaire, including *n* = 138 (21%) GPs and internal specialists as well as *n* = 507 (79%) specialists (*n* = 216 (34%) neurologists, *n* = 190 (30%) ENT specialists, *n* = 101 (16%) urologists). The survey was approved by the Ethical Committee of the Chamber of Physicians of Mecklenburg-Western Pomerania [registry number (BB 127/21)].

### Observation period

The utilization and provision of healthcare services and recognized diseases were captured separately for each month from march 2019 to September 2021. According to Schilling et al. (17), the pandemic timeframe was subdivided into the following periods: 1st-COVID-19-Wave (March–May 2020), Summerplateau 2020 (June–September 2020), 2nd-COVID-19-Wave (October 2020–February 2021) 3rd-COVID-19-Wave (March–June 2021), Summerplateau 2021 (June–July 2021) and 4th-COVID-19-Wave (August–September 2021).

### Study outcomes

#### Outcomes of the secondary data analysis

The main outcomes of the secondary data analysis to illustrate changes in health service utilization during the COVID-19 pandemic were the following: the number of (1) GPs and specialist consultations (telephone contacts and visits), (2) hospital admissions, (3) specialist referrals, and (4) recognized diseases. The following documented ICD-10 diagnoses were used to represent the recognition of diseases in the different practices: dementia (F01, F03, G30 and F06. 7), diabetes mellitus (E10–14), stroke (I63, I64, G45), epilepsy (G40), Parkinson's disease (G20, G21), depression (F32, F33), cancer (C00–C99), chronic bronchitis and chronic obstructive pulmonary disease (COPD; J42–J44), and myocardial infarction (MI; I21, I22) and coronary artery disease (CAD; I24, I25). All diagnoses must be the initial diagnosis. Healthcare utilization and disease recognition were presented as frequencies whereby the pandemic periods (2020 and 2021) were compared with the corresponding prepandemic periods in 2019. For this purpose, percentage differences were calculated for both the individual waves and overall pandemic course compared to the respective prepandemic period to assess the change during the COVID-19 pandemic in Germany.

#### Survey outcomes

The subsequent survey for the practitioners elicited the outpatient physicians' perspectives covering the following outcomes: (1) perceived causes for the decreased and non-compensated number of consultations and recognized diseases during the 1st-COVID-19-wave and the following waves, and (2) the changes made in practice management to reduce infection risk. To assess physicians' perceptions in more detail, two to five additional items assess on a 5-Point-Likert-type response scale (1= “does not apply” to 5= “applies”) whether the physicians thought that themselves or the patient caused the decrease or lack of compensation. Changes in practice management comprise eight items assessing whether the practices changed their management (1= “does not apply” to 5= “applies”) and 11 items assessing how often items were performed compared to pre-pandemic (1= “much rarer” to 5= “much more frequently”). Each of the six items represented measures for infection risk reduction and was assessed dichotomously (yes vs. no). In addition, sociodemographic characteristics of the physicians (age, sex) and the following characteristics of their practices were assessed: state of working (self-employed vs. employed), outpatient facility (solo practice vs. community health center), diagnostic alignment (yes vs. no), number of patients per quarter (0–1.000 vs. 1.001–1.500 vs. >1.500) and contact with designated COVID-19 patients (never vs. rarely vs. often vs. very often). The questionnaire is demonstrated in Supplementary Appendix 1.

### Statistical analyses

Regarding primary and secondary data analysis, descriptive statistics were used. Group differences in proportions of sociodemographic and practice characteristics of the physicians, as well as in perception and views on causes, were determined using Fisher's exact Tests (GPs vs. Specialists). The reporting of the results followed the STROBE guidelines (18). Analyses were performed using SAS version 9.4 (Cary, NC: SAS Institute Inc) and STATA/IC 16 (19).

## Results

### Change in the provision of healthcare services

After an initial sharp decrease in the consultation rate during the first COVID-19 wave, GPs and internists (+2.6) have increased their consultations over the entire COVID-19 pandemic (2020–2021) in comparison to the pre-pandemic period (2019). However, the consultation rate for specialists was reduced (−1.7%) at the end of the fourth COVID-19 wave in September 2021. Neurologists and psychiatrists (+0.9%), urologists (+0.1%) and gynecologists (+3.3%) compensated for an initial consultation rate decrease, while orthopedists (−4.3%), dermatologists (−3.1%) and ENT specialists (−7.7%) had a decreased consultation rate at the end of 2021.

**Figure 1:**
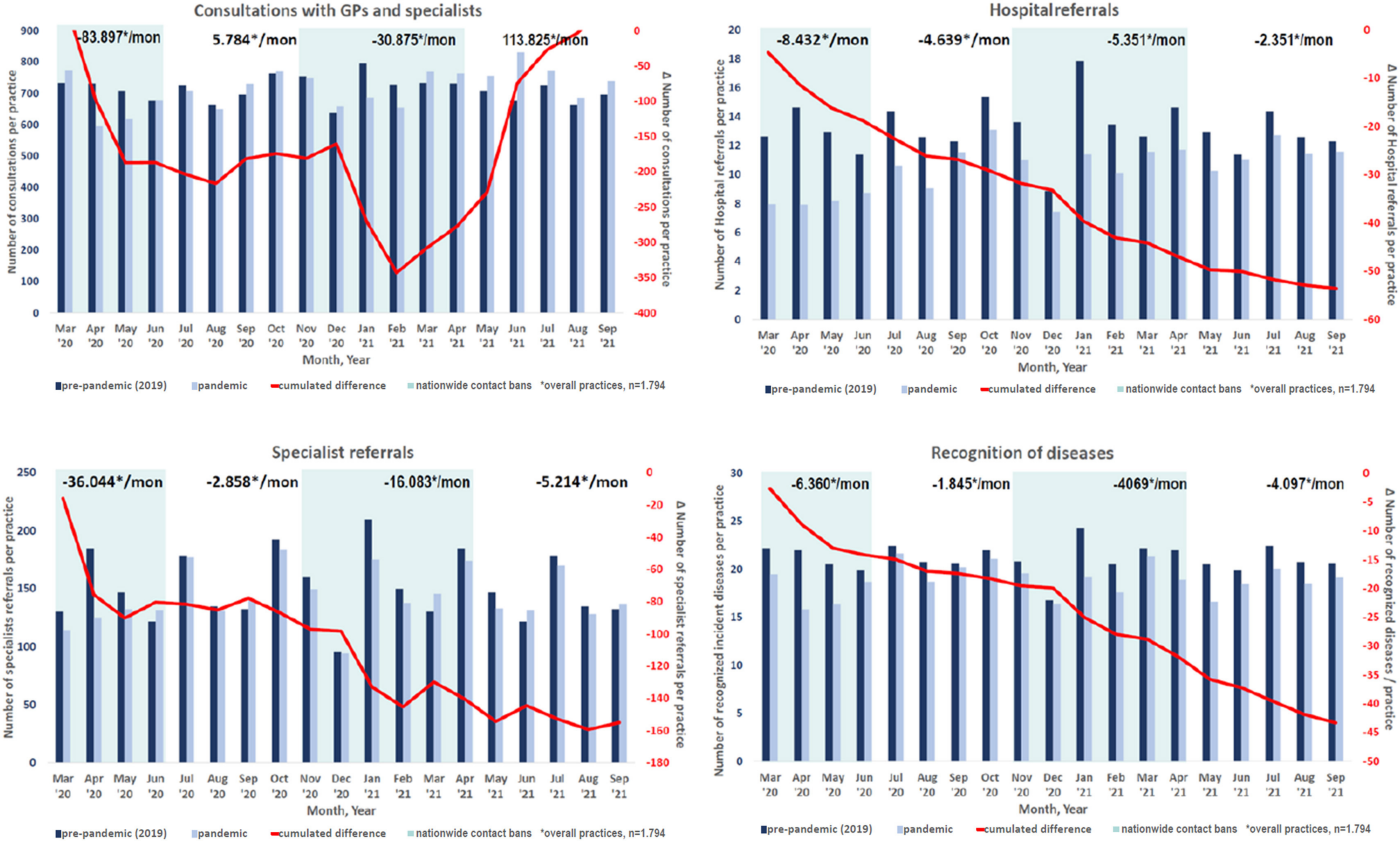
Change of consultation rate, specialist referrals, hospital admissions and detection of diseases over the COVID-19 pandemic.

Over all COVID-19-waves, the specialists' referrals decreased in all practices, especially in GPs and less in specialist practices (−6.0 vs. −2.6%). The largest decrease was seen for ENT specialists (−6.1%). Hospital admissions decreased tremendously over the entire COVID-19 pandemic for both GPs (−21.5%) and specialists (−15.7%). While dermatologists (−32.7%) have demonstrated the largest, urologists (−11.1%) had the lowest decline. Figure 1 and Table 1 show the change in the consultation, specialists' referrals and hospital admission rate for each practitioner specialty over the entire COVID-19 pandemic. A detailed description of these changes can be found in Supplementary Appendix 2.

**Table 1:** Utilization of healthcare services during the COVID-19 pandemic in Germany compared to the pre-pandemic time frame 2019/20.

**Utilization of healthcare services**	**1**^**st**^**-COVID-19-wave**^a^ **(March–May 2020)**			**Summerplateau 2020**^a^ **(June–September 2020)**			**2**^**nd**^**-COVID-19-wave**^a^ **(October 2020–February 2021)**			**3**^**rd**^**-COVID-19-wave**^a^ **(March–June 2021)**			**Summerplateau 2021**^a^ **(June–July 2021)**			**4**^**th**^**-COVID-19-wave**^a^ **(August–September 2021)**			**∑(%)**
**Numbers (*n*)**	**2019**	**2020**	**Diff**.	**2019**	**2020**	**Diff**.	**2019/20**	**2020/21**	**Diff**.	**2019**	**2021**	**Diff**.	**2019**	**2021**	**Diff**.	**2019**	**2021**	**Diff**.	
**in thousands**			**(%)**			**(%)**			**(%)**			**(%)**			**(%^a^)**			**(%)**	
**Number of consultations**																			
GPs & Int. med. (*n* = 996)	2, 422.0	2, 256.7	−6.8	3, 074.2	3, 058.4	−0.5	4, 100.9	3, 932.7	−4.1	3, 156.2	3, 533.7	12.0	1, 560.9	1, 840.6	17.9	1, 513.3	1, 615.8	6.8	+2.6
Total specialists (*n* = 798)	1, 519.0	1, 307.0	−14.0	1, 905.7	1, 903.9	−0.1	2, 502.7	2, 376.2	−5.1	1, 958.5	2, 061.1	5.2	948.1	1, 036.2	9.3	957.6	941.6	−1.7	−1.7
Orthopedists (*n* = 147)	352.1	288.6	−18.0	444.2	440.8	−0.8	575.1	537.4	−6.5	452.6	458.8	1.4	219.4	231.0	5.3	224.8	215.0	−4.4	−4.3
Psychiatrists/Neurol. (*n* = 127)	202.5	196.5	−3.0	261.8	266.4	1.7	340.7	334.8	−1.7	265.6	277.4	4.4	131.4	140.5	7.0	130.4	128.9	−1.2	+0.9
Urologists (*n* = 84)	155.0	136.4	−12.0	191.7	197.0	2.8	259.7	250.9	−3.4	198.4	212.4	7.1	93.8	104.2	11.1	98.0	96.6	−1.4	+0.1
Dermatologists (*n* = 83)	222.4	187.5	−15.7	277.6	275.1	−0.9	356.0	339.0	−4.8	287.2	297.2	3.5	140.0	145.2	3.7	137.5	132.9	−3.4	−3.1
ENT specialists (*n* = 133)	237.5	180.0	−24.2	277.9	269.7	−2.9	382.2	323.4	−15.4	300.8	297.5	−1.1	138.6	154.4	11.4	139.3	137.6	−1.2	−7.7
Gynecologists (*n* = 224)	349.5	317.9	−9.0	452.6	455.0	0.5	589.1	590.7	0.3	453.9	517.8	14.1	224.9	260.8	16.0	227.7	230.8	1.4	+3.3
**Specialists referrals**																			
GPs & Int. med. (*n* = 996)	577.9	447.1	−22.6	684.8	699.1	2.1	979.9	900.1	−8.1	711.7	698.0	−1.9	365.8	364.0	−0.5	319.1	312.6	−2.0	−6.0
Total specialists (*n* = 798)	264.7	219.4	−17.1	332.2	340.2	2.4	459.2	427.8	−6.8	333.3	350.0	5.0	165.5	176.7	6.7	166.6	162.7	−2.3	−2.6
Orthopedists (*n* = 147)	73.5	58.1	−20.9	91.2	93.5	2.5	121.6	113.1	−7.0	92.4	93.9	1.6	45.7	48.3	5.7	45.5	45.2	−0.5	−3.8
Psychiatrists/Neurol. (*n* = 127)	18.6	14.9	−20.2	22.5	22.4	−0.3	31.3	29.1	−7.1	23.2	23.9	2.9	11.5	12.5	8.9	11.0	11.4	3.6	−3.4
Urologists (*n* = 84)	31.3	26.6	−14.8	38.4	40.9	6.6	52.3	52.2	−0.2	39.9	43.9	10.1	18.4	21.3	15.4	20.0	19.9	−0.5	2.3
Dermatologists (*n* = 83)	36.1	30.7	−15.0	44.1	45.3	2.8	60.9	58.3	−4.3	45.6	48.2	5.6	22.1	23.6	6.8	22.0	21.2	−3.8	−1.5
ENT specialists (*n* = 133)	26.9	19.7	−27.0	30.9	32.1	3.6	44.4	40.7	−8.4	33.2	32.7	−1.6	15.8	16.1	2.0	15.2	15.1	−0.3	−6.1
Gynecologists (*n* = 224)	78.3	69.4	−11.3	105.1	106.0	0.8	148.7	134.4	−9.6	98.9	107.3	8.5	52.0	54.9	5.5	53.1	50.0	−5.8	−2.6
**Hospital admissions**																			
GPs & Int. med. (*n* = 996)	46.8	25.7	−45.1	58.4	42.9	−26.5	69.1	58.2	−15.7	59.5	49.7	−16.5	29.6	26.7	−9.8	28.8	26.2	−9.0	−21.5
Total specialists (*n* = 798)	27.2	17.5	−35.9	33.6	28.8	−14.2	43.0	37.0	−14.0	34.2	30.4	−11.2	16.7	15.9	−4.8	16.9	15.1	−10.5	−15.7
Orthopedists (*n* = 147)	7.2	4.8	−33.2	8.8	8.1	−7.9	11.8	10.2	−13.5	9.0	8.1	−10.7	4.3	4.0	−6.0	4.5	4.2	−6.6	−13.6
Psychiatrists/Neurol. (*n* = 127)	3.1	1.8	−42.6	3.9	3.0	−22.9	4.7	3.8	−19.4	4.0	3.3	−16.3	2.0	1.8	−9.5	2.0	1.7	−11.4	−21.3
Urologists (*n* = 84)	4.8	3.2	−32.5	5.7	5.3	−7.1	7.5	6.6	−12.5	6.0	5.6	−5.6	2.8	3.0	8.2	2.9	2.6	−9.4	−11.1
Dermatologists (*n* = 83)	1.3	0.7	−50.9	1.8	1.2	−33.4	2.0	1.5	−28.6	1.7	1.2	−26.3	0.9	0.6	−32.3	0.9	0.6	−25.4	−32.7
ENT specialists (*n*= 133)	4.8	2.7	−43.2	5.7	5.0	−11.6	7.9	6.2	−21.4	5.9	5.0	−15.5	2.8	2.6	−6.8	2.8	2.5	−10.8	−19.5
Gynecologists (*n* = 224)	6.0	4.3	−29.1	7.7	6.2	−19.8	9.0	8.7	−3.3	7.6	7.1	−6.7	3.9	3.8	−2.2	3.9	3.4	−11.6	−12.1

a According to the classification of Schilling et al. (17).

### Recognized diseases

During the COVID-19 pandemic, the number of recognized diseases decreased among GPs and specialist practices by −8.4% (GPs: −9.0% vs. specialists: −7.5%). The largest decline for GPs and internists accounted for COPD (−20.5%), Parkinson's disease (−13.0%) and CAD (−10.5%), followed by depression (−7.8%) and dementia (−7.1%). Acute strokes (−12.1%), diabetes (−10.4%), MI (−9.3%) and cancer (−8.5%) experienced the largest decrease among specialists. Table 2 demonstrates the change in recognized diseases. A detailed description of the changed recognition of diseases over time is also demonstrated in Supplementary Appendix 2.

**Table 2:** Recognition of diseases during the COVID-19 pandemic in Germany compared to the pre-pandemic time frame 2019/20.

**Recognized disease**	**1**^**st**^**-COVID-19-wave**^a^ **(March–May 2020)**			**Summerplateau 2020**^a^ **(June–September 2020)**			**2**^**nd**^**-COVID-19-wave**^a^ **(October 2020–February 2021)**			**3** ^**rd**^ **-COVID-19-wave (March–June 2021)**			**Summerplateau 2021**^a^ **(June–July 2021)**			**4**^**th**^**-COVID-19-wave**^a^ **(August–September 2021)**			**∑(%)**
**Numbers (*n*)**	**2019**	**2020**	**Diff**.	**2019**	**2020**	**Diff**.	**2019/20**	**2020/21**	**Diff**.	**2019**	**2021**	**Diff**.	**2019**	**2021**	**Diff**.	**2019**	**2021**	**Diff**.	
**in thousands**			**(%)**			**(%)**			**(%)**			**(%)**			**(%^a^)**			**(%)**	
**All diagnoses**	114.8	93.1	−18.9	146.7	142.1	−3.1	181.7	168.4	−7.3	147.2	135.0	−8.3	73.4	69.1	−5.9	73.3	67.5	−7.9	−8.4
**GPs. Int. med. special. (*****n*** **=** **996)**	70.6	58.1	−17.7	90.8	87.4	−3.7	111.3	103.3	−7.1	90.8	81.3	−10.5	45.9	41.7	−9.2	44.9	41.7	−7.0	−9.0
Diabetes	15.3	12.0	−21.7	19.6	19.2	−2.2	23.4	23.0	−1.7	19.8	19.1	−3.4	10.1	10.1	0.1	9.5	9.8	2.7	−4.7
Cancer	9.5	7.9	−16.8	13.0	12.6	−3.1	15.2	14.4	−5.1	12.2	11.6	−4.8	6.6	6.1	−8.1	6.4	6.0	−6.4	−6.8
Coronary aertery disease	8.2	6.5	−21.3	10.7	10.2	−4.3	12.5	12.0	−4.0	10.7	9.0	−16.3	5.5	4.8	−12.0	5.3	4.8	−8.4	−10.5
Dementia	4.4	3.8	−15.2	6.1	6.3	3.6	7.4	7.1	−3.8	5.7	4.9	−13.7	3.0	2.6	−11.7	3.1	2.9	−7.3	−7.1
COPD	8.7	8.1	−6.9	9.2	8.1	−12.0	14.6	10.0	−31.1	10.7	7.7	−28.6	4.7	3.7	−21.1	4.5	4.1	−10.1	−20.5
Depression	17.2	13.4	−21.9	22.3	21.3	−4.2	27.0	25.6	−5.2	22.2	20.8	−6.4	11.1	10.7	−3.8	11.1	10.4	−6.4	−7.8
Acute stroke	3.4	3.0	−12.2	4.6	4.6	−0.3	5.4	5.4	−0.2	4.4	4.1	−7.2	2.3	2.0	−11.7	2.3	2.2	−3.7	−5.0
Myocardial infarction	1.3	1.4	2.2	1.8	1.7	−3.7	2.0	2.0	−0.5	1.8	1.3	−26.6	0.9	–	–	0.9	–	–	−7.6
Parkinson	1.0	0.8	−22.3	1.4	1.3	−11.3	1.5	1.4	−2.3	1.3	1.0	−19.9	0.7	0.6	−13.9	0.7	0.7	−7.1	−13.0
Epilepsy	1.5	1.3	−14.0	2.1	2.1	−1.4	2.4	2.4	−0.5	1.9	1.8	−8.6	1.0	1.0	−6.1	1.1	1.0	−8.8	−5.3
**All specialists (*****n*** **=** **798)**	44.2	35.1	−20.6	55.9	54.7	−2.2	70.4	65.0	-7.7	56.4	53.7	−4.8	27.5	27.4	−0.5	28.4	25.7	−9.5	−7.5
Diabetes	7.7	5.7	−26.0	9.6	9.3	−3.0	12.1	10.7	−11.5	9.8	9.1	−7.4	4.7	4.5	−5.0	4.9	4.4	−9.0	−10.4
Cancer	14.2	11.1	−21.5	18.3	17.8	−2.4	23.0	20.5	−10.7	18.1	17.1	−5.7	8.9	9.1	1.7	9.3	8.3	−10.4	−8.5
Coronary heart dis.	2.1	1.6	−22.7	2.6	2.6	−1.1	3.4	3.0	−11.4	2.6	2.6	−0.8	1.3	1.3	0.4	1.4	1.3	−8.9	−7.7
Dementia	3.0	2.4	−22.1	4.0	4.0	−0.3	5.1	4.6	−9.7	3.9	4.0	4.1	2.0	2.1	4.5	2.0	1.9	−3.6	−5.0
COPD	1.4	1.2	−10.6	1.7	1.7	1.5	2.2	2.0	−7.5	1.7	1.7	−1.5	0.8	0.8	5.3	0.9	0.8	−9.5	−4.0
Depression	11.4	9.1	−20.5	13.9	13.5	−2.4	17.7	16.9	−4.5	14.5	13.7	−5.5	6.9	6.9	−0.4	6.9	6.3	−8.5	−6.9
Acute stroke	1.6	1.2	−24.9	2.1	1.9	−8.9	2.4	2.2	−8.1	2.1	1.8	−13.7	1.0	1.0	−4.2	1.1	1.0	−12.1	−12.1
Myocardial infarction	0.3	0.3	−8.6	0.3	0.3	−8.4	0.4	0.4	14.2	0.4	0.2	−35.7	0.2	–	–	0.2	–	–	−9.3
Parkinson	1.2	1.0	−21.6	1.6	1.6	1.8	2.1	1.8	−11.2	1.6	1.6	−0.5	0.8	0.8	5.0	0.9	0.7	−13.6	−6.8
Epilepsy	1.3	1.6	18.5	1.8	1.9	2.8	2.2	2.8	30.5	1.7	1.9	8.4	0.9	0.9	2.3	0.9	0.9	0.1	12.7

a According to the classification of Schilling et al. (17).

### Practitioners' perception and causal reasons for the change in the healthcare provision and utilization

Table 3 summarizes the physicians' and practices' characteristics. The substantial decrease in primary care and specialist consultations was perceived by almost all physicians (81%), but especially in GPs practices (92 vs. 77%, *p* = 0.001). GPs and specialists surveyed saw the reasons for the decline primarily in changes in patient behavior (97% agreement). In contrast, less than half (48%) of physicians subjectively perceived the decline of the detection of diseases during the first lockdown - again, significantly more in GPs practices than in specialists' practices (56 vs. 43%, *p* = 0.027), also self-justified by changes in the patient behavior (97% agreement). In contrast, only 40% of physicians perceived a decline in the further course of the pandemic. Again, changed patient behavior (agreement in 95% of cases) was often considered the causative reason. Sixty-four perent of physicians, especially GPs (83 vs. 59%, *p* = 0.024), indicated the continued high burden, and the additional services such as vaccinations and corona testing prevented compensatory effects (75 vs. 42%, *p* = 0.001). Table 4 summarizes the survey results.

**Table 3:** Characteristics of the physicians and their practices.

	**Total sample**	**GPs, Int. med**.	**ENT specialists**	**Psychiatrists/Neurolog**.	**Urologists**	**Total specialists**	***p*** **-Value^‡^**
	***n*** **= 645**	**special. *n* = 138**	***n*** **= 190**	***n*** **= 216**	***n*** **= 101**	***n*** **= 507**	
**Age group**, ***n*** **(%)**
< 35	3 (0.47)	2 (1.45)	1 (0.53)	–	–	1 (0.20)	0.114
35–44	72 (11.16)	14 (10.14)	30 (15.79)	20 (9.26)	8 (7.92)	58 (11.44)	
45–65	512 (79.38)	114 (82.61)	142 (74.74)	172 (79.63)	84 (83.17)	398 (78.50)	
>65	58 (8.99)	8 (5.80)	17 (8.95)	24 (11.11)	9 (8.91)	50 (9.86)	
**Sex**, ***n*** **(%)**
Female	229 (35.67)	82 (59.85)	66 (34.92)	69 (32.09)	12 (11.88)	147 (29.11)	**< 0.001**
**State of working**, ***n*** **(%)**
Self-employed	613 (95.19)	134 (97.81)	178 (93.68)	202 (93.52)	99 (98.02)	479 (94.48)	0.119
Employed	31 (4.81)	3 (2.19)	12 (6.32)	15 (6.48)	2 (1.98)	28 (5.52)	
**Outpatient facility** ***n*** **(%)**
Solo practice	361 (57.67)	96 (71.64)	99 (54.70)	114 (54.03)	52 (52.00)	265 (53.86)	**< 0.001**
Community health center	265 (42.33)	38 (28.36)	82 (45.30)	97 (45.97)	48 (48.00)	227 (46.14)	
**Diagnostic alignment**, ***n*** **(%)**
Yes	268 (42.14)	35 (25.93)	118 (62.77)	55 (25.82)	60 (60.00)	233 (46.51)	**< 0.001**
No	368 (57.86)	100 (74.07)	70 (37.23)	158 (74.1)	40 (40.00)	268 (53.49)	
**Number of patients per quarter**, ***n*** **(%)**
0–1.000	150 (23.36)	38 (27.74)	20 (10.53)	82 (38.32)	10 (9.90)	112 (22.18)	0.103
1.001–1.500	174 (27.10)	42 (30.66)	44 (23.16)	58 (27.10)	30 (29.70)	132 (26.14)	
>1.500	318 (49.53)	57 (41.61)	126 (66.32)	74 (34.58)	61 (60.40)	261 (51.68)	
**Contact with COVID-19 patients**, ***n*** **(%)**
Never	55 (8.55)	1 (0.73)	11 (5.79)	34 (15.81)	9 (8.91)	54 (10.67)	**< 0.001**
Rarely	365 (57.77)	17 (12.41)	118 (62.11)	149 (69.30)	81 (80.20)	348 (68.77)	
(Very) often	223 (34.68)	119 (86.86)	61 (32.11)	32 (14.88)	11 (10.89)	104 (20.55)	

‡ Differences in proportions (GPs, Int. med. special. vs. Specialists): Fisher's exact test for categorical variables and Kruskal-Wallis test for ordinal variables.

Values in bold indicate *p* < 0.05.

**Table 4:** Perception and Causes from the outpatient physicians' perspective.

	**Total sample**		**GPs, Int. med. special**.		**Specialists**		***p*** **-Value^‡^**
	***N***	***n*** **(%)**	***N***	***n*** **(%)**	***N***	***n*** **(%)**	
**Perceived decrease in consultation during the 1** ^**st**^ **-COVID-19-Wave (ref. yes)**	484	390 (81%)	103	95 (92%)	381	295 (77%)	**0.001**
The causes were changes in appointment allocation and practice organization^*^
*Does (rather) not apply*	363	210 (58%)	92	53 (58%)	271	157 (58%)	1.000
*Applies (rather)*		134 (37%)		34 (37%)		100 (37%)	
The cause was a change in patient behavior^*^
*Does (rather) not apply*	384	11 (3%)	93	5 (5%)	291	6 (2%)	0.224
*Applies (rather)*		372 (97%)		88 (95%)		284 (98%)	
**Perceived decrease in incidence during the 1** ^**st**^ **-COVID-19-Wave (ref. yes)**	464	231 (46%)	94	53 (56%)	370	160 (43%)	**0.027**
The causes were changes in appointment allocation and practice organization^*^
*Does (rather) not apply*	196	118 (60%)	49	22 (45%)	147	96 (65%)	**0.037**
*Applies (rather)*		54 (28%)		19 (39%)		35 (24%)	
The cause was a change in patient behavior^*^
*Does (rather) not apply*	210	5 (2%)	53	1 (2%)	157	4 (3%)	0.331
*Applies (rather)*		204 (97%)		51 (96%)		153 (97%)	
The cause was the postponement of appointments until after the lockdown^*^
*Does (rather) not apply*	204	25 (12%)	52	4 (8%)	152	21 (14%)	0.550
*Applies (rather)*		168 (82%)		45 (87%)		123 (81%)	
Video and telephone consultations have made detection more difficult^*^
*Does (rather) not apply*	165	63 (38%)	48	19 (40%)	117	44 (38%)	0.068
*Applies (rather)*		67 (41%)		14 (29%)		53 (45%)	
COVID protection measures have made detection more difficult^*^
*Does (rather) not apply*	155	51 (33%)	43	15 (35%)	112	36 (32%)	0.854
*Applies (rather)*		81 (52%)		21 (49%)		60 (54%)	
**Perceived lack of compensation of recognized diseases during the following waves (ref. yes)**	458	185 (40%)	88	36 (41%)	370	149 (40%)	0.905
The causes were changes in appointment allocation and practice organization^*^
*Does (rather) not apply*	171	106 (62%)	35	20 (57%)	136	86 (63%)	0.761
*Applies (rather)*		51 (30%)		12 (34%)		39 (29%)	
The cause was a change in patient behavior^*^
*Does (rather) not apply*	180	6 (3%)	36	2 (6%)	144	4 (3%)	0.642
*Applies (rather)*		171 (95%)		34 (94%)		137 (95%)	
The cause was the persistently high load^*^
*Does (rather) not apply*	173	39 (23%)	35	5 (14%)	138	34 (25%)	**0.024**
*Applies (rather)*		111 (64%)		29 (83%)		82 (59%)	
The cause was the provision of additional services (e.g., vaccinations)^*^
*Does (rather) not apply*	164	61 (37%)	36	4 (11%)	128	57 (45%)	**0.001**
*Applies (rather)*		81 (49%)		27 (75%)		54 (42%)	
The reason for this was the increasing incidence of mental illness^*^
*Does (rather) not apply*	169	27(16%)	35	1 (3%)	134	26 (19%)	**0.038**
*Applies (rather)*		125 (74%)		31 (89%)		94 (70%)	

* Missing percentage values are assigned to the category “neither”.

‡ Differences in proportions (GPs, Int. med. special. vs. Specialists): Fisher's exact Tests.

Values in bold indicate *p* < 0.05.

### Change in practice management in primary care and specialist practices

Nearly half of the participating physicians stated that they reduced home visits (36%), nursing home visits (41%) and opening hours of the practice (40%) during the pandemic. This was seen more frequently in GP than in specialist practices. Thirty-eight percent and 43% of physicians reported that preventive checkups were suspended or non-urgent examinations canceled, respectively, more likely by GPs than specialists (46 vs. 36%, *p* = 0.044). 71% of surveyed GPs and specialists reported that appointments for high-risk patients for a more severe COVID-19 course were more likely canceled. GPs were also significantly more likely to use remote consultation (84 vs. 64%, *p* = 0.001). 80% of GPs also reported a provision of specialist prescriptions or specialist care in place of the specialists. Additionally, more than half of physicians (58%) experienced corona-related staff absences, and one in four practices were temporarily closed due to the absence and illnesses of practice personnel. Moreover, 62 and 40% of practices established a separate waiting area outside or within the practice. The changes in the practice segment are shown in Table 5.

**Table 5:** Change in practice management in primary care and specialist practices.

	**Total sample**		**GPs, Int. med. special**.		**Specialists**		***p*** **-Value^‡^**
	***N***	***n*** **(%)**	***N***	***n*** **(%)**	***N***	***n*** **(%)**	
	**Applies (rather)**		**Applies (rather)**		**Applies (rather)**		
Home visits reduced	433	159 (36%)	112	56 (50%)	316	101 (32%)	**0.001**
Nursing home visits reduced	429	177 (41%)	86	36 (42%)	338	139 (41%)	0.903
Appointments strongly reduced	532	214 (40%)	113	56 (50%)	410	153 (37%)	**0.023**
Office hours strongly reduced	530	93 (18%)	113	26 (23%)	409	63 (15%)	0.066
Appointments for high-risk patients reduced	533	377 (71%)	110	83 (75%)	414	287 (69%)	0.240
External diagnostics used due to infection risk	519	52 (10%)	109	13 (12%)	401	37 (9%)	0.467
Organization of urgent surgeries was challenging	390	181 (46%)	89	37 (41%)	292	138 (47%)	0.395
Organization of elective surgeries was challenging	395	284 (72%)	90	66 (73%)	296	210 (71%)	0.691
	**(much) more frequently**		**(much) more frequently**		**(much) more frequently**		
Preventive checkups suspended	456	174 (38%)	110	51 (46%)	346	123 (36%)	**0.044**
Non-urgent examinations canceled	497	214 43%	107	51 (47%)	382	157 (41%)	0.226
Home visits reduced	407	165 (41%)	107	51 (48%)	300	112 (37%)	**0.036**
Nursing home visits reduced	406	170 (42%)	82	35 (43%)	321	134 (42%)	0.901
Tele and video consultations used	416	282 (68%)	88	74 (84%)	320	204 (64%)	**0.001**
Practice closing	370	88 (24%)	80	18 (23%)	284	68 (24%)	0.882
Employee absences	462	269 (58%)	96	64 (67%)	359	200 (56%)	0.062
Material shortage	504	387 (77%)	104	79 (76%)	391	301 (77%)	0.896
Patients referred directly to specialist	355	30 (8%)	108	8 (7%)	243	22 (9%)	0.684
Substitute specialist prescription	364	184 (51%)	111	89 (80%)	244	93 (38%)	**0.001**
Substitute specialist care	360	172 (48%)	110	81 (74%)	243	90 (37%)	**0.001**
	**Applies (rather)**		**Applies (rather)**		**Applies (rather)**		
Separate waiting area inside the practice	550	221 (40%)	116	53 (46%)	434	168 (39%)	0.201
Separate waiting area outside the practice	550	340 (62%)	116	81 (70%)	434	259 (60%)	**0.053**
COVID-19 specific hygiene concept for practice	550	482 (88%)	116	104 (90%)	434	378 (87%)	0.528
Triage - separation of patient flows	550	225 (41%)	116	90 (78%)	434	135 (31%)	**0.001**
Use air ventilation system	550	234 (43%)	116	46 (40%)	434	188 (43%)	0.529
Training of personnel	550	416 (76%)	116	91 (78%)	434	325 (75%)	0.467

^‡^Differences in proportions (GPs, Int. med. special. vs. Specialists): Fisher's exact Tests. Values in bold indicate *p* < 0.05.

## Discussion

This analysis provided valuable evidence in healthcare services utilization and recognition of diseases in primary and specialized care during the COVID-19 pandemic and tried to illustrate physicians' perceived causes for the changes. The analysis elicited significant differences in the impact of the COVID-19 pandemic on the provision of routine care between primary and specialized practitioners. While GPs and internists could economically compensate the consultation rate over the entire pandemic due to additional services such as COVID-19 vaccinations, most specialists remained on a lower consultation rate. Specialist referrals, hospital admissions, and disease recognition decreased tremendously across all physicians but were stronger among GPs than specialists. Physicians perceived the decline in consultations but not the decrease in disease recognition. Nevertheless, reasons for the observed changes in healthcare utilization and disease recognition were seen by physicians in the changed patient behavior, especially in the postponement or cancellation of appointments during the COVID-19-related lockdowns. However, this study also revealed significant changes in practice management, like reducing nursing home visits and general home visits, shortened practice opening hours, suspended checkups and delayed consultations for high-risk patients. These changes could also impact the revealed changes in the healthcare provision, especially the detection of diseases.

Several studies have evaluated the impact of the early COVID-19 pandemic on primary care consultations, representing a dramatic decline (9, 11, 13, 14, 20). This analysis confirmed this decrease in outpatient consultations, especially at the beginning of the pandemic during the nationwide contact bans. However, the utilization of healthcare services provided by practitioners could partially be compensated over the further course. Schäfer et al. (13) found the effect was independent of specialty during the early stage of the pandemic, which is not in line with the results of this analysis, revealing tremendous differences between physician specializations. While GPs, internists, neurologists, urologists, and gynecologists could increase their consultations over the pandemic, orthopedists, dermatologists, and ENT specialists had a consultation rate deficit. For GPs and some other physician specializations, the upturn of the consultation rate correlated with the number of COVID-19 vaccinations given in primary and specialized care practices (21). The assumption of increased consultation rates due to COVID-19-related vaccinations is supported by the different perceptions of GPs and specialists. Moreover, prescription, hospital admission, specialists' referral, and disease detection rates did not compensate as the consultations did. On the contrary, these important rates decreased when the practices started with the vaccination. However, future studies need to confirm this assumption of increased COVID-19 vaccination-driven consultations and the coinciding occurrence of continuous negative trends regarding the other rates.

Additionally, the survey confirmed that the tremendous decline of consultations during the 1st-COVID-19-wave was perceived by the GPs (92%) and specialists (77%). However, while a patient-centered survey by Schuster et al. (22) revealed that the share of patient-initiated cancellations of primary care appointments was smaller than healthcare-initiated, only 37% of outpatient physicians surveyed in this study agreed that the decline could be traced back to the provider behavior, whereas 97% saw the cause in the changed patient behavior. Furthermore, recent studies support the thesis that the patient's behavior could be reasonable for the delay of outpatient service utilization regarding preventive care and visits for chronic diseases due to anxiety, reduced social activities, increased infection risk and a lack of perception of non-disabling symptoms (20, 23–26). The surveyed physicians supported these indications, confirming (97%) reasons for declined disease recognition remain in changed patient behavior. Moreover, 82% agreed with the cause that the postponement of appointments leads to this decrease. However, according to Bitzer et al. (27), patients lacked decision support to seek routine care during the COVID-19 pandemic, which could affect the demand for consultations under social distancing conditions. Further patient-centered research is, therefore, needed to evaluate the divergence and minimize the barriers to healthcare access in pandemic crises.

Further studies have already revealed a decrease in specialist referrals, hospital admissions and disease recognition during the first month of the COVID-19 pandemic (11, 13). The present analysis was in line with these findings. While the reduced number of hospital admissions was indicated by the limited number of beds in intensive care units, the decline in specialist referrals, especially by GPs and internists, could suggest a breach of the gatekeeping function usually associated with appropriate referral for specialty care (28, 29). This suggestion could be underlined by the not compensated decrease in disease recognition, which is also aligned with the literature, especially for diagnoses such as COPD, several types of cancer, dementia, acute stroke, CAD, and MI (23, 26, 30–33). However, not even half (46 and 40%) of physicians noticed this decline in recognition of diseases. A closer look at each physician's practice reveals that although these declines represent an enormous societal dimension, there were only −11.2 fewer diagnoses per GP practice and −6.4 fewer diagnoses per specialist practice over the entire pandemic. Therefore, whether individual practices or physicians could have perceived these decreases is questionable. These figures could likely be below a “perception threshold.” Alerting physician practices, indicating a significant declining trend, could be vital to impose countermeasures that aim to counteract the countervailing trend demonstrating a non-maintenance of routine primary care during pandemic times. Such solutions could be IT-based systems providing necessary information. Thus, further research about such interventions and the long-term impact of a reduced or delayed detection of diseases are highly relevant.

### Limitations

It was not feasible to assess the extent to which emergency, urgent, and deferrable services were provided within the available diagnostic categories, limiting the assessment of whether individual physician practices properly prioritized diagnostic and treatment strategies in light of the high-risk situation and lockdown. While the reported results and discussion primarily comprise the summarized results over the entire study period, in-depth analyses of differences within waves were not the focus of this analysis. Further research is needed to highlight the differences between the respective stages of the pandemic, with greater attention to the various diagnoses and differences between age groups. Moreover, findings are related to treatment cases. Therefore, results do not allow conclusions to be drawn at the individual patient level because patient behavior was not captured in the available data.The primary data gained in the survey followed a cross-sectional design, limiting causal conclusions. In addition, the data is not representative of the statistical population of all physicians in Germany, especially regarding sociodemographic characteristics or the regional distribution, respectively. Furthermore, selection bias should be considered since data was collected *via* an online survey distributed by professional associations of specializations through their respective communication channels. The survey includes retrospectively asked questions drawn upon participants' memories. Therefore, possible recall bias should also be taken into account.

## Conclusion

The COVID-19 pandemic significantly impacted primary and specialized care. Whereas specialist referral, hospital admission, and the diseases recognition rate steadily decreased over the COVID-19 pandemic, the initial decline in the physician consultation rate could be compensated at the end of 2021, probably due to additional service provisions such as COVID-19 vaccinations. GPs specialist referrals and hospital admissions also decreased much stronger than in specialist practices. Although most physician practices perceived the initial sharp decline of routine care, the number of non-detected diseases in each practice compared to the years before could be too minor for recognition in routine care practice. Physicians have seen patient behavior as the major contributor to this decline, comprising appointment cancellations or lacked decision support to seek routine care under social distancing conditions. However, changes in practice management may also have affected the tremendous decline in consultation, referrals, hospital admissions, and disease recognition. Mitigating the pandemic while maintaining routine care represents a major challenge for healthcare systems worldwide. Thus, decision-makers should divide responsibilities to ensure continued access to routine primary and specialty care on the one hand and to mitigate pandemics on the other. Furthermore, organizational and financial support for GPs and specialists' practices is urgently needed to prevent long-term adverse effects on patient outcomes.

## Data availability statement

The original contributions presented in the study are included in the article/Supplementary material, further inquiries can be directed to the corresponding author.

## Ethics statement

The present study was reviewed and approved by the Ethical Committee of the Chamber of Physicians of Mecklenburg-Western Pomerania [Registry Number (BB 127/21)]. The patients/participants provided their written informed consent to participate in this study.

## Author contributions

All authors listed have made a substantial, direct, and intellectual contribution to the work and approved it for publication.

## Conflict of interest

Author KK was an employee of IQVIA. The remaining authors declare that the research was conducted in the absence of any commercial or financial relationships that could be construed as a potential conflict of interest. The reviewer AS declared a shared parent affiliation with the author JB to the handling editor at the time of review.

## Publisher's note

All claims expressed in this article are solely those of the authors and do not necessarily represent those of their affiliated organizations, or those of the publisher, the editors and the reviewers. Any product that may be evaluated in this article, or claim that may be made by its manufacturer, is not guaranteed or endorsed by the publisher.
