# Supplementary material for: The long-term impact of the COVID-19 pandemic on primary and specialized care provision and disease recognition in Germany
Source: Front Public Health. 2022 Nov 17;10:1006578. doi: 10.3389/fpubh.2022.1006578 (PMC9712961; doi:10.3389/fpubh.2022.1006578)
Supplement: Supplementary file 1 [file Data_Sheet_1.PDF]

Appendix 1

Information about yourself and your medical office: In the first section we would like to know something about you and your medical office. We therefore ask fort he following information:

**Information about your medical office:**				
**Postal code:**	I_I_I_I_I_I			
**Type of medical office:**	☐ Single practice	☐ Joint practice	☐ Medical Care Center	
**Amount of patients treated per quarter:**	☐ 0 – 500	☐ 501 – 1000	☐ 1001 – 1500	☐ >1500
**We predominantly treat patients with...**	☐ chronic diseases	☐ acute diseases	☐ both	
**Our medical office is predominantly diagnostically oriented.**	☐ yes	☐ no		
**Information about yourself and your professional practice:**				
**Age group:**	☐ <35	☐ 35 – 44	☐ 45 – 65	☐ >65
**Sex:**	☐ male	☐ female	☐ diverse	
**Employment relationship:**	☐ self-employed	☐ employed		
**Which professional association do you belong to?**	☐ General Practioners Association Berlin and Brandenburg e.V	☐ German Orthopedic & Trauma Surgeons Association e.V	☐ Professional Association of German Neurologists e.V.	
	☐ German Professional Association of Otolaryngologists e.V		☐ Professional Association of German Urologists e.V	
**Frequency of contact with patientes tested positive for Covid-19 throughout the pandemic:**	☐ never	☐ rarely	☐ often	☐ very often

Changes in practice management and practice activities: In the following section we would like to know something about, how much your organisation of your medical office has changed during the COVID-pandemic. Please indicate which statement applies to you, based on the time of the pandemic.

	**Does not apply**	**Rather not true**	**neither**	**Rather true**	**Applies**
I have reduced home visits to patients.	☐	☐	☐	☐	☐
I have reduced nursing home visits.	☐	☐	☐	☐	☐
I have greatly reduced the amount of call-ins due to Covid-19 and postponed non-essential appointments.	☐	☐	☐	☐	☐
I have reduced the office hours and associated tasks due to Covid-19.	☐	☐	☐	☐	☐
COVID-19 at-risk patients (elderly, multimorbid) were less likely to visit the practice in person during the pandemic.	☐	☐	☐	☐	☐
Because of the risk of infection, I used external diagnostics (e.g. radiology, hospital) more frequently for clarification.	☐	☐	☐	☐	☐
It was challenging to organize appointments for…					
Urgent operations	☐	☐	☐	☐	☐
Elective operations	☐	☐	☐	☐	☐
nformation and recommendations on the design of the practice organization (e.g. by the Association of Statutory Health Insurance Physicians, Ministry, Health Department, etc.)					
Came in time	☐	☐	☐	☐	☐
Were sufficient & target-oriented.	☐	☐	☐	☐	☐

Compared to 2019, during the Covid 19 pandemic, how often did you ...	Much more often	More often	Remained the same	Less frequenty	Much less frequently
...not perform preventive examination, with the aim to detecting certain diseases?	☐	☐	☐	☐	☐
...cancel non-urgent examinations?	☐	☐	☐	☐	☐
...reduce home visits to patients?	☐	☐	☐	☐	☐
...reduce nursing home visits?	☐	☐	☐	☐	☐
...conduct telephone and/or video consultations?	☐	☐	☐	☐	☐
...have to close your practice?	☐	☐	☐	☐	☐
...have to handle employee absence (e.g., Home-Schooling)?	☐	☐	☐	☐	☐
...have to suffer from shortages of infection control materials (e.g., masks or gowns)?	☐	☐	☐	☐	☐
...refer patients directly to a specialist?	☐	☐	☐	☐	☐
...take over specialist prescriptions for patientson a proxy basis?	☐	☐	☐	☐	☐
... take over specialist care on a for patients proxy basis?	☐	☐	☐	☐	☐

What specific protective measures have you taken as a result of COVID-19? Please tick them off (multiple answers possible):

☐ *Separate waiting areas (Covid vs. Non-Covid patients.)*
☐ *Waiting area outside the office*
☐ *Protective clothing and protective material for practice personnel (respirators, disposable gloves, etc.)*
☐ *Covid-19-specific practice-specific hygiene concept*
☐ *Triage of patients/separation of patient flows.*
☐ *Ventilation system*
☐ *Training/ further education of practice personnel*
☐ *Other:* ______________________

Reasons for and consequences of the change in practice activities: Scientific analyses of medical documentation in physicians' offices indicated that there were significant changes in practice patterns during and especially at the beginning of the pandemic. This related to the frequency of consultations and the number of newly detected (incidence) chronic diseases. If you think back again: Did you notice these changes and what do you think were the causes?

During the 1st lockdown (April/ May 2020), the analyses showed a decrease in the frequency of consultations. Have you noticed this decline in your practice? What do you think were possible causes for the decline in your practice?

Consultation decline perceived in practice: ☐ yes / ☐ no	Does not apply	Rather not true	neither	Rather true	Applies
This was due to changes in call-in behavior and practice organization (expansion of telephone and video consultation hours).	☐	☐	☐	☐	☐
This was due to changes in patient behavior (appointment cancellation, absenteeism).	☐	☐	☐	☐	☐

The cause was the following other reasons:

____

____

Analyses showed that fewer new cases of chronic diseases were detected during the 1st lockdown (April/ May 2020). Have you noticed this decline in your practice? What do you think were possible causes for the fewer new cases detected in your practice?

Incidence decline perceived in practice : ☐ yes / ☐ no	Does not apply	Rather not true	neither	Rather true	Applies
This was caused by changes in the call-in behavior and practice organization .	☐	☐	☐	☐	☐
This was due to changes in patient behavior (appointment cancellation, absenteeism).	☐	☐	☐	☐	☐
Postponement of appointments until after the lockdown has negatively affected the detection rate.	☐	☐	☐	☐	☐
More commonly used digital treatment channels via telephone or video have made detection more difficult.	☐	☐	☐	☐	☐

COVID-related protection meassurements have made reliable detection difficult.	☐	☐	☐	☐	☐

The cause was the following other reasons:

_ _

_ _

Until the 2nd lockdown as well as during the 2nd lockdown (waves 2 and 3), the decline in detected new cases could not be completely compensated for. Have you noticed this lack of compensation? What do you think were possible causes in your practice?

Lack of compensation perceived in practice : ☐ yes / ☐ no	**Does not apply**	**Rather not true**	**neither**	**Rather true**	**Applies**
This was caused by changes in the call-in behavior and practice organization .	☐	☐	☐	☐	☐
This was due to changes in patient behavior (appointment cancellation, absenteeism).	☐	☐	☐	☐	☐
The cause was the persistently high burden.	☐	☐	☐	☐	☐
The cause was the provision of additional services (e.g. vaccination)	☐	☐	☐	☐	☐
This was due to the increasing incidence of mental illnesses (e.g. anxiety disorders, depression)	☐	☐	☐	☐	☐

The cause was the following other reasons:

_ _

_ _

Appendix 2: Detailed description of changes of primary and specialized care provision and disease recognition in primary and specialized care

Consultation rate

During the 1st-Covid-19-wave, the consultation rate decreased in all practices, with the most intensive decrease in ENT physicians (-24%) and the smallest decrease in psychiatrists and neurologists (-3%). During the summer plateau of 2020, psychiatrists and neurologists (+2%), urologists (+3%) and gynecologists (+1%) demonstrated an increasing consultation rate compared to 2019, while the consultation rate continuously decreased for all other practitioner practices. Except for gynecologists (+0,3%), the consultation rate decreased in all practices during the 2nd-Covid-19-wave. Again, ENT specialists (-15%) had the largest, and psychiatrists and neurologists (-2%) had the smallest decrease. All practices increased their number of consultations, entering the 3rd-Covid-19-wave at the beginning of 2021, except the ENT specialists (-1%). Gynecologists (+14%) demonstrated the strongest increase, followed by GPs and internists (+12%). For all practices, the consultation rate increased during the summer plateau of 2021. However, this increase continues only for Gynecologists (+1%), GPs and internists (+7%) in the 4th-Covid-19-wave.

Specialists' referrals

During the 1st-Covid-19-wave, specialist referrals decreased in all practices. ENT specialists (-27%) had the strongest, and gynecologists (-11%) had the lowest decline. Except for neurologists and psychiatrists (-0,3%), specialist referrals increased during the summer plateau of 2020. All practices decreased their referrals to other specialists entering the 2nd-Covid-19-wave at the end of 2020, while gynecologists (-10%), ENT specialists (-8%) and GPs and internists (-8%) accounted for the highest decline. Most practices referred more frequently to other specialists during the 3rd-Covid-19-wave than in the same time before the pandemic, except for ENT specialists (-2%) and GPs and internists (-2%). While the specialists' referrals increased during the summer plateau of 2021, they decreased during the 4th-Covid-19-wave, except for neurologists and psychiatrists (+4%).

Hospital admissions

The decrease in hospital admissions during the 1st-Covid-19-wave amounts to -51% for dermatologists. They still have the largest decline during the summer plateau of 2020 (-33%). The pattern of decline was maintained for the next Covid-19-waves and did not change until the summer plateau of 2021 when at least Urologists (+8%) demonstrated an increase in hospital admissions. The values decreased again over the respective practices entering the 4th-Covid-19-wave.

Recognized incident disease

During the 1st-Covid-19-wave, the percentage change ranged between -22% (Parkinson's disease) and -7% (COPD) among GPs and internists. Specialists document -26% fewer diabetes, -25% fewer acute strokes and -23% fewer CAD diagnoses than in the same time frame before the pandemic. While after the first wave, except for dementia (+4%), the trend still declines among the GPs and internists, other specialists increased their recognition of epilepsy (+3%), Parkinson's disease (+2%) and COPD (+2%). For the next two COVID waves, the GPs and internists documented up to -29% fewer COPD, -27% fewer MI and -20% fewer Parkinson's disease diagnoses than before the pandemic. At the same time, specialists increased their recognized incident disease to +8% for epilepsy and +4% for dementia. While they expanded these tendencies and increased the number of documented diagnoses except for diabetes (-5%) and acute strokes (-4%) during the summer plateau of 2021, the GPs and internists confirmed the negative trend of the previous waves (up to -21% COPD, -14% Parkinson's disease, -12% dementia, -12 CAD). For both, the number of recognized incident diseases declined again, entering the 4th-Covid-19-wave.
